# Assessment of oral health status and related factors in adolescents aged 12–15 years in the Gansu Province of China: a cross-sectional survey

**DOI:** 10.1186/s12903-023-02748-y

**Published:** 2023-01-25

**Authors:** Zhidong Zhang, Di Wang, Jian Zhao, Dandan Wang, Baoping Zhang

**Affiliations:** 1 School of Stomatology, Lanzhou University, Lanzhou, 730000 China; 2 Hospital of Stomatology Lanzhou University, Lanzhou, 730000 China; 3 Gansu Province Key Lab of Maxillofacial Reconstruction and Intelligent Manufacturing, Lanzhou, 730000 China; 4 Institute of Biomechanics and Medical Engineering, Lanzhou University, Key Laboratory of Mechanics on Disaster and Environment in Western China, Ministry of Education, Lanzhou University, Lanzhou, 730000 China

**Keywords:** Adolescents, Surveys and questionnaires, Oral health, Cross-sectional study, China

## Abstract

**Background:**

The national oral epidemiological survey conducted every decade has become an indispensable means of detecting changes in oral disease patterns. This study was undertaken to investigate the oral health status and related factors in 12–15-year-old students in Gansu, China.

**Methods:**

According to the methodology adopted by the Fourth National Oral Health Survey, a multi-stage, stratified, random sampling method was used to select 3871 adolescents aged 12–15 years from four regions of Gansu Province for oral examination and questionnaire survey. Caries experience was measured using the Decayed, Missing, and Filled Teeth (DMFT) index; and periodontal health examination included gingival bleeding, calculus, periodontal pockets and attachment loss. The questionnaire included questions regarding sociodemographic characteristics, and oral health knowledge and behaviors. SPSS20.0 software was used for statistical analysis of the survey data.

**Results:**

The mean DMFT index was 0.83 ± 1.42. The prevalence of caries experience was 38.6%, filling rate was 1.6%, and pit and fissure sealing rate was 0.5%. Logistic regression analysis showed that female sex, rural district, older age, non-only child, frequency of dental visits, and toothache experience were the risk factors for caries experience, with OR ranging between 1.280 and 3.831 (*p* < 0.05). Prevalence of healthy periodontium was 29.8%. Female sex, rural district, and younger age were found to be the protective factors for healthy periodontium, with OR ranging between 1.178 and 1.414 (*p* < 0.05).

**Conclusions:**

Adolescents in Gansu Province had high prevalence of caries experience along with low filling rate, and low prevalence of healthy periodontium. Therefore, it is necessary to vigorously strengthen oral health education, disease prevention and control programs in the province. This would help improve the oral health-related quality of life of these individuals.

**Supplementary Information:**

The online version contains supplementary material available at 10.1186/s12903-023-02748-y.

## Background

According to the 2017 Global Burden of Disease Report, approximately 3.5 billion people were affected by oral diseases, of which dental caries and periodontitis were the two most commonly occurring entities [1]. Dental caries is the most common chronic disease among children and young adults [2]. It has been reported to affect 60–90% of pre-school children and almost 100% of adults worldwide [3]. It is also a common and frequently-occurring disease among adolescents. Periodontal disease is a chronic inflammatory disease of the periodontium caused by plaque microorganisms. Poor oral hygiene is a well-known and important risk factor for periodontal diseases. Middle school students aged 12–15 years undergo transition from mixed to permanent dentition. This critical period determines the oral health status in adulthood, and is also the pivotal stage of establishment of oral health concepts and behaviors [4]. Therefore, investigation of oral health status in this population has important significance for oral health maintenance.

Disparities in occurrence of dental caries in different regions of the country reflect the uneven geographical distribution of the number of dental caries (DMFT index) [5]. Therefore, analyzing the oral epidemiological survey data of different topographical regions of China would be of great significance in formulating corresponding preventive measures to improve the overall oral health. Gansu province is located in the western part of China, and has the most complex geographical environment in the country. Diet of the people of this province mainly comprises noodles, and is supplemented by coarse grains. Moreover, they have a taste for hot and sour food. The province consists of several minorities, and the dietary structure is relatively complex. Dental caries has been reportedly correlated with several factors like socioeconomic status, level of education, dietary habits, and geographical environment [6, 7]. Therefore, studying caries-related factors in Gansu Province, a place with varied regional characteristics, would have unique advantages.

With the development of China's social economy and improvement in people's living standards in the recent years, the diet structure has undergone tremendous change, and new changes have emerged in oral health [8, 9]. Previous studies have shown that the prevalence of dental caries in 12-year-old children in China increased from 28.9% in 2005 to 38.5% in 2015 [10, 11]. According to the latest oral health survey in China, gingival bleeding and calculus affected more than 60% of the adolescent population [12]. Therefore, this study was undertaken to investigate the latest epidemiological characteristics of oral health status of adolescents in Gansu province. We assessed the oral health status and related factors among adolescents aged 12–15 years in Gansu province, with the intent to provide a basis for local governments to develop realistic and effective policies for disease prevention and health care.

## Methods

### Sampling

Middle school students of Gansu Province, aged 12–15 years formed our study population.

The following formula was used to calculate the sample size:

$$n = deff\frac{{\mu_{\alpha }^{2} p(1 - p)}}{{\delta^{2} }}.$$

where *deff* was the sampling design effect set as 4.5, *p* was the caries rate of permanent teeth in the 12-year-old age group (28.9% as reported by the Third National Oral Health Survey in 2005), the margin of error of *p* was controlled at 10%, *μ* was the level of confidence and *α* was set to 0.05 [13, 14]. Based on this estimation, the theoretical sample size was calculated to be 3840.

Multi-stage, stratified random sampling was used to select the study participants. In the first stage, two districts (Qinzhou District Tianshui City, Kongtong District Pingliang City) and two counties (Li County Longnan City, Zhenyuan County Qingyang City) in Gansu Province were randomly sampled using probability-proportional-to-size sampling (PPS). In the second stage, three junior schools in each county (district) were selected using the PPS method. In the last stage, 320 students aged 12–15 years in each junior school were randomly selected using quota sampling method. Thus, the final sample comprised 3871 students.

Oral epidemiological survey was conducted among adolescents aged 12–15 years in Gansu Province between 2015 and 2016. This study protocol was approved by the Ethics Committee of Chinese Stomatological Association and the Ethics Committee of School of Stomatology Lanzhou University (No. 2014–003). Informed consent was obtained from all the study participants before the commencement of the study. If participants were under the age of 18 years, consent was obtained from a parent and/or legal guardian. All procedures were carried out in accordance with relevant guidelines and regulations.

### Clinical examination and questionnaire survey

The oral checklist and questionnaire were formulated in accordance with the Fourth National Oral Health Survey Program and Basic Methods of Oral Health Survey (5th Edition) designed by World Health Organization (WHO) [8, 13]. Oral examination was performed using a plane mouth mirror and a Community Periodontal Index (CPI) probe under artificial light source. It comprised combination of inspection and probing, and included evaluating the crown and periodontal condition of permanent teeth. The 12-, 13-, and 14-year-old age groups were assessed for gingival bleeding and calculus, while the 15-year-old age group was evaluated for gingival bleeding, calculus, periodontal pockets, and attachment loss.

The oral examination indicators and scoring methods were shown in Table 1. The results of dental caries were described by the number of decayed, missing and filled teeth (DMFT). DMFT = 0 was recorded as no caries, while DMFT ≥ 1 was recorded as caries experience [14, 15]. Absence of gingival bleeding, calculus, deep periodontal pockets and attachment loss ≤ 3 mm were the criteria for healthy periodontium [12].

**Table 1:** Oral examination indicators and scoring methods

Examination indicators	0	1	2	3	4	5	6	9	X
Crown caries	No	Crown caries	Crown caries with filling teeth	No crown caries with filling teeth	Missing for caries	Missing for other reasons	Pit and fissure sealing	N/A	–
Gingival bleeding	No	Gingival bleeding	–	–	–	–	–	N/A	Missing
Calculus	No	Calculus	–	–	–	–	–	N/A	Missing
Periodontal pockets	No	4~5 mm	≥ 6 mm	–	–	–	–	N/A	Missing
Attachment loss	0~3 mm	4~5 mm	6~8 mm	9~11 mm	≥ 12 mm	–	–	N/A	Missing

*N/A* no records

The questionnaire [13] mainly included questions regarding sociodemographic characteristics (gender, age, urban or rural areas, only child or not), frequency of consumption of sweets (rarely, 1 to 4 times a month, 2 to 7 times a week, ≥ 2 times a day), time since their last dental visit (within 6 months, 6–12 months, more than 12 months, never), toothache experience (often, occasional, can’t remember, never), and oral health knowledge (poor, average, good, excellent).

### Quality control

All the dental examiners were stomatology graduates with the qualifications of dental practitioners and more than three years of clinical work experience. Before conducting on-site investigation work, all the examiners received technical training for theoretical and dental examination. They also mastered the investigation plan and examination technology. Before the survey, each inspector received the standard consistency test of caries status and periodontal pocket depth inspection. Only when the *Kappa* values of caries status and periodontal pocket depth reached 0.8 and 0.6 or more, respectively, they could participate in the survey.

### Statistical analysis

SPSS 20.0 software was used for statistical analyses. Measurement data was expressed as mean ± standard deviation, and comparison of means was performed using *t* test (two samples) and one-way ANOVA (three or more samples). Enumeration data was expressed as percentages, and analyzed using Chi-square test. Multivariate logistic regression analysis was used for analyzing the associated factors. The test standard was set at *p* < 0.05.

## Results

The *Kappa* value range of caries status and periodontal pocket depth among dental examiners in Gansu Province were 0.91–1.00 and 0.68–0.73, respectively [13]. A total of 3871 students aged 12–15 years were assessed in this study. Of these, 3848 were included in the study after data quality control screening. The final study population consisted of 930 students in the 12 years old group, 966 in 13 years old group, 990 in 14 years old group, and 962 in 15 years old group (Table 2).

**Table 2:** Distribution of district and gender among a sample of 12–15 years old in Gansu province of China (n = 3848)

Characteristics	12 years old	13 years old	14 years old	15 years old
Urban				
Female	236	244	257	240
Male	231	232	256	243
Total	467	476	513	483
Rural				
Female	229	248	238	241
Male	234	242	239	238
Total	463	490	477	479

The prevalence of caries experience was 38.6%, while the filling and pit and fissure sealing rates were 1.6% and 0.5% respectively. The mean DMFT score was 0.83 ± 1.42, which was mainly composed of DT (0.80 ± 1.40) accounting for approximately 96.4%, followed by FT (0.02 ± 0.18) and MT (0.00 ± 0.06) accounting for 2.4% and 1.2%, respectively. The mean DMFT of males and females were 0.73 ± 1.31 and 0.93 ± 1.53 respectively, and the difference was found to be statistically significant (*Z* = − 4.155, *p* < 0.001). Rural students had higher DMFT scores compared to the urban students (*Z* = − 14.127, *p* < 0.001). The mean DMFT was also found to be significantly different among the various age groups (*H* = 27.300, *p* < 0.001). Comparison between groups are detailed in Table 3 and Additional file 1: Fig. S1. The filling and pit and fissure sealing rates differed significantly between the urban and rural districts. The filling rate among urban students (2.1%) was significantly higher compared to rural students (1.1%) (*p* = 0.017). Moreover, pit and fissure sealing rate among urban students (0.9%) was also significantly higher than that among rural students (0.1%) (*p* < 0.001).

**Table 3:** Dental condition among a sample of 12–15 years old in Gansu Province of China (n = 3848)

Characteristics	DT	MT	FT	DMFT	Filling rate %	Pit and fissure sealing rate %
Gender	–	–	–	–	–	–
Female	0.70 ± 1.29	0.00 ± 0.05	0.02 ± 0.18	0.73 ± 1.31	1.9	0.6
Male	0.91 ± 1.50	0.00 ± 0.07	0.02 ± 0.19	0.93 ± 1.53	1.3	0.4
District	–	–	–	–	–	–
Urban	0.45 ± 0.91	0.01 ± 0.07	0.03 ± 0.21	0.49 ± 0.95	2.1	0.9
Rural	1.16 ± 1.70	0.00 ± 0.04	0.02 ± 0.16	1.18 ± 1.71	1.1	0.1
Age (years)	–	–	–	–	–	–
12	0.64 ± 1.15	0.00 ± 0.06	0.01 ± 0.11	0.65 ± 1.17	1.0	0.9
13	0.72 ± 1.25	0.00 ± 0.06	0.02 ± 0.17	0.74 ± 1.27	1.6	0.5
14	0.92 ± 1.51	0.00 ± 0.06	0.02 ± 0.16	0.94 ± 1.52	1.5	0.3
15	0.93 ± 1.61	0.00 ± 0.06	0.04 ± 0.26	0.97 ± 1.64	2.3	0.2
Total	0.80 ± 1.40	0.00 ± 0.06	0.02 ± 0.18	0.83 ± 1.42	1.6	0.5

Chi-square analysis revealed that older female students residing in rural areas, who were not the only child of their parents, frequently consumed sweets, recently had a dental visit and experienced toothache had higher prevalence of caries experience (*p* < 0.05). Inclusion of the above mentioned factors in multivariate logistic regression analysis showed that female sex (odds ratio [OR] = 1.294, 95% confidence interval [CI] = 1.129–1.483, *p* < 0.001), rural district (OR = 2.381, 95%CI = 2.063–2.749, *p* < 0.001), older age (15 years old: OR = 1.562, 95%CI = 1.284–1.901, *p* < 0.001; 14 years old: OR = 1.638, 95% CI 1.349–1.989, *p* < 0.001), non-only child (OR = 1.280, 95% CI 1.004–1.633, *p* = 0.047), dental visits (within 6 months: OR = 2.136, 95% CI 1.529–2.983, *p* < 0.001; 6 ~ 12 months: OR = 1.699, 95% CI 1.287–2.243, *p* < 0.001; over 12 months: OR = 1.627, 95% CI 1.324–2.000, *p* < 0.001), and toothache experience (often: OR = 3.831, 95% CI 2.592–5.661, *p* < 0.001; occasionally: OR = 1.582, 95% CI 1.331–1.881, *p* < 0.001) were the risk factors for caries experience (Tables 4, 5).

**Table 4:** Univariate analysis of caries and periodontal health status among a sample of 12–15 years old in Gansu province of China (Chi-square analysis)

Characteristics	Caries rate			Periodontal health rate		
	Rate %	χ^2^ value	*p* value	Rate %	χ^2^ value	*p* value
Gender	–	13.26	< 0.001	–	5.48	0.019
Female	35.7	–	–	28.0	–	–
Male	41.5	–	–	31.5	–	–
District	–	147.37	< 0.001	–	9.18	0.002
Urban	29.1	–	–	27.5	–	–
Rural	48.2	–	–	32.0	–	–
Age (years)	–	23.89	< 0.001	–	13.75	0.003
12	33.2	–	–	32.8	–	–
13	36.5	–	–	29.5	–	–
14	42.8	–	–	31.3	–	–
15	41.5	–	–	25.5	–	–
Only children	–	10.82	0.001	–	0.832	0.362
Yes	31.0	–	–	31.7	–	–
No	39.5	–	–	29.5	–	–
Frequency of sweets consumption	–	8.36	0.039	–	0.53	0.912
Rarely	31.0	–	–	31.0	–	–
1 to 4 times a month	38.4	–	–	29.9	–	–
2 to 7 times a week	40.3	–	–	29.2	–	–
≥ 2 times a day	40.4	–	–	31.9	–	–
The time of recent dental visit	–	24.70	< 0.001	–	3.09	0.378
Within 6 months	51.5	–	–	27.5	–	–
6 to 12 months	45.9	–	–	34.4	–	–
More than 12 months	42.1	–	–	30.0	–	–
Never	36.6	–	–	29.4	–	–
Toothache experience	–	95.73	< 0.001	–	2.55	0.467
Often	64.5	–	–	28.4	–	–
Occasionally	41.7	–	–	29.2	–	–
Never	29.2	–	–	31.9	–	–
Can’t remember	29.3	–	–	29.0	–	–
Oral health knowledge	–	2.99	0.394	–	18.14	< 0.001
Poor	44.2	–	–	27.3	–	–
Average	36.9	–	–	24.5	–	–
Good	38.3	–	–	29.7	–	–
Excellent	40.3	–	–	34.4	–	–

**Table 5:** Logistic regression analysis of associated factors related to caries status among a sample of 12–15 years old in Gansu province of China

Characteristics	B value	Standard error	Wald value	OR (95% CI)	*p* value
Gender					
Female	0.257	0.070	13.662	1.294 (1.129–1.483)	< 0.001
Male	–	–	–	–	–
District					
Rural	0.868	0.073	140.331	2.381 (2.063–2.749)	< 0.001
Urban	–	–	–	–	–
Age (years)					
15	0.446	0.100	19.811	1.562 (1.284–1.901)	< 0.001
14	0.494	0.099	24.818	1.638 (1.349–1.989)	< 0.001
13	0.136	0.101	1.837	1.146 (0.941–1.396)	0.175
12	–	–	–	–	–
Only children					
No	0.247	0.124	3.956	1.280 (1.004–1.633)	0.047
Yes	–	–	–	–	–
Frequency of sweets consumption					
Rarely	0.099	0.343	0.082	1.104 (0.563–2.164)	0.774
1 to 4 times a month	0.328	0.318	1.063	1.388 (0.744–2.589)	0.303
2 to 7 times a week	0.311	0.320	0.947	1.365 (0.730–2.533)	0.330
≥ 2 times a day	–	–	–	–	–
The time of recent dental visit					
Within 6 months	0.759	0.171	19.788	2.136 (1.529–2.983)	< 0.001
6 to 12 months	0.530	0.142	13.976	1.699 (1.287–2.243)	< 0.001
More than 12 months	0.487	0.105	21.472	1.627 (1.324–2.000)	< 0.001
Never	–	–	–	–	–
Toothache experience					
Often	1.343	0.199	45.426	3.831 (2.592–5.661)	< 0.001
Occasionally	0.459	0.088	27.089	1.582 (1.331–1.881)	< 0.001
Can’t remember	− 0.033	0.105	0.051	0.968 (0.729–1.285)	0.821
Never	–	–	–	–	–

The prevalence of gingival bleeding and calculus were 70.2% and 85.6%, respectively. More number of teeth among male students were found to be associated with gingival bleeding and calculus in comparison to female students(*Z* = − 2.027, *p* = 0.043; *Z* = − 3.974, *p* < 0.001, respectively). No significant difference was observed between urban and rural students in terms of number of teeth associated with gingival bleeding (*Z* = − 0.661, *p* = 0.509). However, urban students had fewer teeth associated with calculus than the rural students (*Z* = − 16.658, *p* < 0.001). The number of teeth associated with gingival bleeding and calculus were found to be significantly different in the different age groups (*H* = 16.146, *p* = 0.001; *H* = 132.224, *p* < 0.001, respectively), as shown in Table 6 and Supplementary Fig. 1. Table 7 shows that the prevalence of periodontal pockets and attachment loss were 12.7% and 2.4% respectively. Additionally, no gender and regional differences were observed in the context of number of teeth associated with periodontal pockets and attachment loss.

**Table 6:** Incidence of gingival bleeding and calculus among a sample of 12–15 years old in Gansu province of China (n = 3848)

Characteristics	Gingival bleeding		Calculus	
	Means	Rate %	Means	Rate %
Gender	–	–	–	–
Female	5.69 ± 6.84	71.9	7.54 ± 6.35	86.4
Male	5.24 ± 6.43	68.5	6.69 ± 5.79	84.9
District	–	–	–	–
Urban	5.24 ± 6.12	72.5	5.54 ± 5.06	80.8
Rural	5.69 ± 7.13	67.9	8.71 ± 6.61	90.5
Age (years)	–	–	–	–
12	4.93 ± 5.88	67.2	5.45 ± 5.00	78.9
13	5.33 ± 6.48	70.5	6.83 ± 5.93	86.2
14	5.41 ± 6.98	68.7	7.60 ± 6.38	87.0
15	6.17 ± 7.07	74.4	8.51 ± 6.49	90.1
Total	5.46 ± 6.64	70.2	7.11 ± 6.09	85.6

**Table 7:** Periodontal pockets and attachment loss among a sample of 15 years old in Gansu province of China (n = 962)

Characteristics	Periodontal pockets		Attachment loss	
	Means	Rate %	Means	Rate %
Gender	–	–	–	–
Female	0.31 ± 1.07	12.9	0.04 ± 0.30	2.5
Male	0.27 ± 0.95	12.5	0.05 ± 0.35	2.3
District	–	–	–	–
Urban	0.33 ± 1.06	15.1	0.05 ± 0.37	2.5
Rural	0.24 ± 0.97	10.2	0.04 ± 0.28	2.3
Total	0.29 ± 1.01	12.7	0.04 ± 0.33	2.4

The prevalence of healthy periodontium was 29.8%. Chi-square analysis showed that young female students residing in rural areas had higher prevalence of healthy periodontium (*p* < 0.05). Inclusion of the above factors in multivariate logistic regression analysis showed that female sex (OR = 1.178, 95% CI 1.025–1.355, *p* = 0.021), rural district (OR = 1.316, 95% CI 1.141–1.519, *p* < 0.001), and younger age (12 years old: OR = 1.414, 95% CI 1.156–1.730, *p* = 0.001; 13 years old: OR = 1.207, 95% CI 0.987–1.478, *p* = 0.067; 14 years old: OR = 1.339, 95% CI 1.097–1.635, *p* = 0.004) were the protective factors for healthy periodontium (Tables 4, 8).

**Table 8:** Logistic regression analysis of associated factors related to periodontal health status among a sample of 12–15 years old in Gansu province of China

Characteristics	B value	Standard error	Wald value	OR (95% CI)	*P* value
Gender					
Female	0.164	0.071	5.289	1.178 (1.025–1.355)	0.021
Male	–	–	–	–	–
District					
Rural	0.275	0.073	14.179	1.316 (1.141–1.519)	< 0.001
Urban	–	–	–	–	–
Age (years)					
12	0.347	0.103	11.355	1.414 (1.156–1.730)	0.001
13	0.189	0.103	3.345	1.207 (0.987–1.478)	0.067
14	0.292	0.102	8.236	1.339 (1.097–1.635)	0.004
15	–	–	–	–	–
Oral health knowledge					
Excellent	0.320	0.269	1.410	1.377 (0.812–2.335)	0.235
Good	0.136	0.263	0.267	1.146 (0.684–1.920)	0.605
Average	− 0.106	0.274	0.149	0.900 (0.526–1.538)	0.700
Poor	–	–	–	–	–

## Discussion

This study aimed to investigate the oral health status and related factors in 12–15-year-old students in Gansu Province of China to identify and solve the oral health-related problems, and to improve the quality of life of the surveyed population. The prevalence of caries experience was found to be 38.6%, which was similar to that reported in China (38.5%) [13]. However, this rate was slightly higher compared to Brazil (37%) and Norway (37%), and slightly lower than Poland (42%) [16].

Female students had significantly higher prevalence of caries experience than males did, which was consistent with the survey findings of Zhejiang and Jiangxi provinces [14, 17]. Some studies have pointed out that females are more prone to caries in comparison to males due to the following factors: teeth eruption occurs earlier in females, and comparatively slower saliva flow due to fluctuation of hormones during puberty [18]. This may also be related to girls’ preference for cariogenic foods. Univariate analysis in the present study and other studies have shown that the prevalence of caries experience was higher among individuals who frequently consumed sweet food items [19–21]. The prevalence of caries experience in rural district was found to be significantly higher compared to urban district. This finding was in concordance with the results of the survey on trends of dental caries in permanent teeth among 12-year-old children in China between 1995 and 2014, and the Fourth National Oral Healthy Survey [21, 22]. This may be related to lack of treatment and oral health knowledge in rural areas. It was observed that the incidence of caries increased with age, however, the pit and fissure sealing rate decreased with increase in age. A large number of studies have shown that sealing of pit and fissures can effectively prevent caries [23, 24], but the pit and fissure sealing rate among adolescents aged 12–15 years in Gansu province was only 0.2–0.9%, which was far lower than the sealing rate of 5.7% among adolescents in China [13]. Therefore, it is necessary to take appropriate steps to strengthen oral health education, especially among rural and female adolescents. Moreover, the coverage of oral disease intervention programs such as pit and fissure sealing should be more widespread to reduce the prevalence of dental caries.

It was observed that only children had lower prevalence of caries experience, which was in accordance with the finding of a study which examined the oral health of only and non-only children in China [25]. This may be because parents pay more attention to the oral health of their only child. In addition, dental visit experience was found to be closely related to occurrence of dental caries [21]. A study reported that people with toothache experience had higher incidence of dental caries [26]. Once an individual has oral health problems such as toothache, they immediately seek medical attention to treat the condition, reduce further incidence of caries and prevent the occurrence of other oral diseases.

The prevalence of gingival bleeding and calculus in adolescents aged 12–15 years in the Gansu province were 70.2% and 85.6% respectively, which were significantly higher compared to the same age group in China (61.0% and 67.3%) [13]. This indicated that the periodontal health status of adolescents in Gansu province was poor. The periodontal health status of females was found to be significantly better than males, and this significant gender difference has also been reported by other adolescents' periodontal health status surveys [27–29]. This may be related to the fact that females pay more attention to health and oral hygiene [30]. The prevalence of healthy periodontium of urban students was lower than that of rural students. Data from the Fourth National Oral Health Survey showed that the periodontal status of adolescents in rural areas of eastern China was significantly better than the urban areas of western China, which may have been related to difference in dietary structure and living habits [27].

WHO recommends the age of 15 years for evaluating the prevalence of periodontitis among adolescents [8, 13]. The results of the present survey showed that the detection rates of periodontal pockets and attachment loss in 15-year-old students were 12.7% and 2.4% respectively, which were significantly higher than the detection rates of these two entities during the same period in China (6.5% and 0.5%) [13]. In addition, a large number of studies [27, 31] have reported that younger the age, better was the periodontal condition, which was consistent with the findings of the present study. Therefore, diagnosis, treatment and prevention of periodontal diseases should be started from early adolescence.

The concept of prevention first and combination of prevention and treatment has gradually assimilated in the lives of people. Dental caries can be prevented using population strategies that result in behavioral changes [32]. Techniques based on behavior change to reduce the incidence of dental caries were limited, and focused primarily on information regarding disease aetiology and hygiene habits [33]. Also, combined interventions would prove to be more effective, since isolated oral health education interventions did not improve oral health over time. Moreover, interventions carried out by dentists at school that include health education and individualized oral hygiene have been reported to be effective in reducing dental caries in permanent teeth [34]. Therefore, local medical institutions need to further strengthen oral health advocacy, especially in economically underdeveloped rural areas. Oral health knowledge should be actively promoted and regular oral examinations for adolescents aged 12–15 years must be carried out to reduce the incidence of oral diseases and improve the oral health-related quality of life of students.

The present study analyzed the oral health status of 12–15-year-old adolescents of Gansu province in accordance with the data of the Fourth National Oral Epidemiological Survey of Gansu Province. A large sample size was obtained using multi-stage stratified random sampling method, and the results were reliable, which provided the current basis for oral health management of 12–15-year-old adolescents in Gansu Province. However, this study also had few limitations. Relevant variable factors were collected using questionnaires, which inevitably affects the accuracy of results due to recall bias. In addition, DMFT index was used for caries assessment, which only measures the presence or absence of caries. However, the severity of caries remained unassessed. Caries could be small enough to be difficult to detect, or could involve relatively larger area and destroy the entire crown. Therefore, DMFS index can be used in future studies to more accurately reflect the severity of caries epidemic in terms of lesions affecting the tooth surface.

## Conclusions

This study showed that the prevalence of caries experience was high and the prevalence of healthy periodontium was low among adolescents aged 12–15 years in the Gansu Province of China. Moreover, the rates of caries filling and pit closures were also relatively low. Moreover, periodontal disease was more commonly associated with gingival bleeding and calculus. The results of multivariate analysis showed that gender, area of residence (urban or rural), age and other factors were significantly associated with oral health.

## Supplementary Information

**Additional file 1: Fig. S1**. Comparation of the mean DMFT, gingival bleeding and calculus. (a) Comparation of the mean DMFT between male sex and female sex. (b) Comparation of the mean DMFT between urban areas and rural areas. (c) Comparation of the mean DMFT among different ages. (d) Comparation of the mean number of teeth detected by gingival bleeding between male sex and female sex. (e) Comparation of the mean number of teeth detected with gingival bleeding between urban areas and rural areas. (f) Comparation of the mean number of teeth detected with gingival bleeding among different ages. (g) Comparation of the mean number of teeth detected with calculus between male sex and female sex. (h) Comparation of the mean number of teeth detected by calculus between urban areas and rural areas. (i) Comparation of the mean number of teeth detected by calculus among different ages.

## Data Availability

All data generated or analyzed during this study are included in this article.
