# Supplementary figures and images for: Assessment of oral health status and related factors in adolescents aged 12–15 years in the Gansu Province of China: a cross-sectional survey
Source: BMC Oral Health. 2023 Jan 25;23:42. doi: 10.1186/s12903-023-02748-y (PMC9875395; doi:10.1186/s12903-023-02748-y)

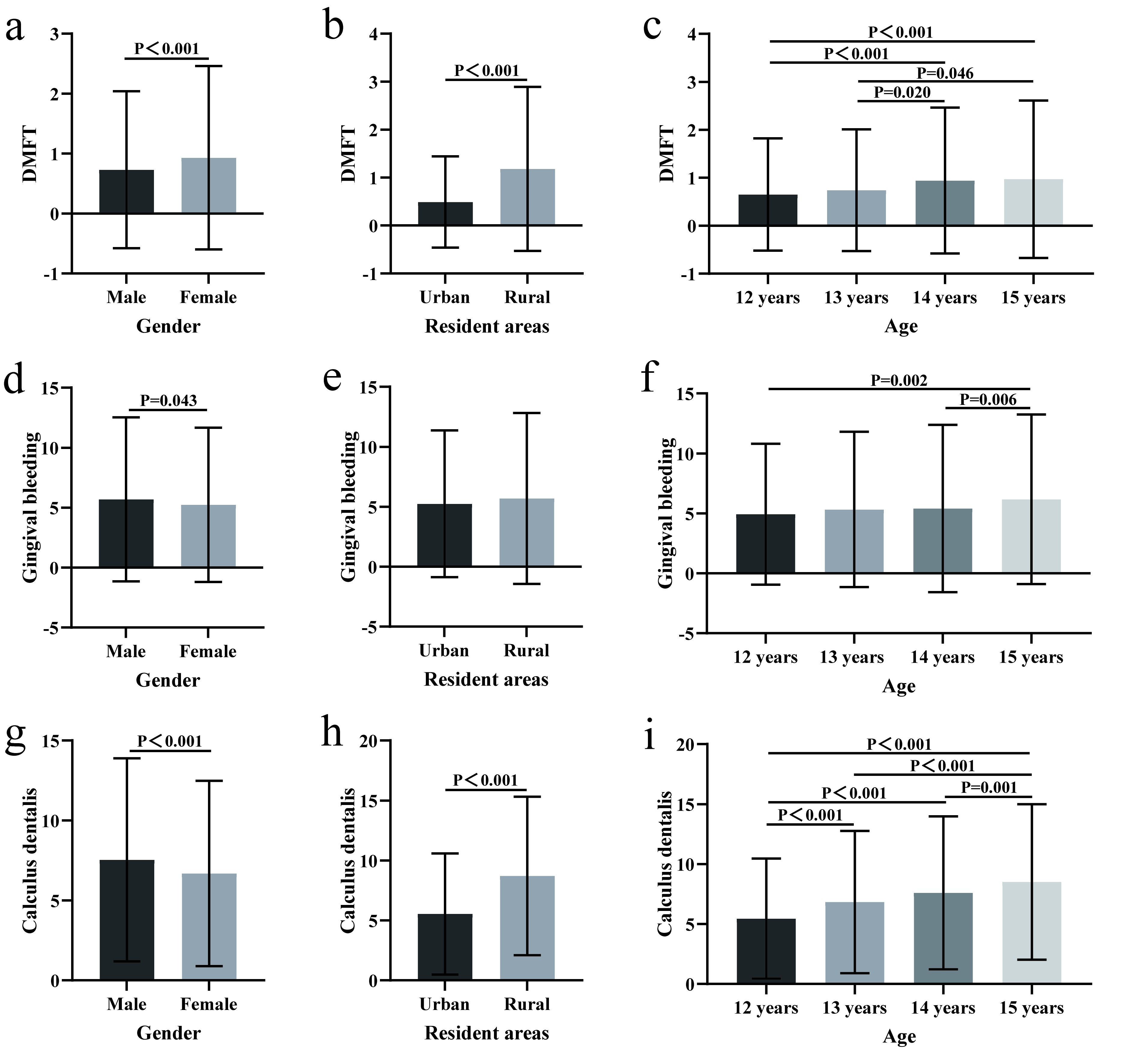

Supplement: Supplementary file 1 — Additional file 1: Fig. S1. Comparation of the mean DMFT, gingival bleeding and calculus. (a) Comparation of the mean DMFT between male sex and female sex. (b) Comparation of the mean DMFT between urban areas and rural areas. (c) Comparation of the mean DMFT among different ages. (d) Comparation of the mean number of teeth detected by gingival bleeding between male sex and female sex. (e) Comparation of the mean number of teeth detected with gingival bleeding between urban areas and rural areas. (f) Comparation of the mean number of teeth detected with gingival bleeding among different ages. (g) Comparation of the mean number of teeth detected with calculus between male sex and female sex. (h) Comparation of the mean number of teeth detected by calculus between urban areas and rural areas. (i) Comparation of the mean number of teeth detected by calculus among different ages. [file 12903_2023_2748_MOESM1_ESM.tif]
